# Recovery of NMDA receptor currents from MK-801 blockade is accelerated by Mg^2+^ and memantine under conditions of agonist exposure

**DOI:** 10.1016/j.neuropharm.2013.01.024

**Published:** 2013-11

**Authors:** Sean McKay, C. Peter Bengtson, Hilmar Bading, David J.A. Wyllie, Giles E. Hardingham

**Affiliations:** a Centre for Integrative Physiology, University of Edinburgh School of Biomedical Sciences, Hugh Robson Building, George Square, Edinburgh EH8 9XD, UK; b Department of Neurobiology, Interdisciplinary Center for Neurosciences (IZN), Im Neuenheimer Feld 364, D-69120 Heidelberg, Germany

**Keywords:** Glutamate receptor, NMDA receptor, Pharmacology, Synapse, Extrasynaptic, Excitotoxicity, MK-801, Magnesium

## Abstract

MK-801 is a use-dependent NMDA receptor open channel blocker with a very slow off-rate. These properties can be exploited to ‘pre-block’ a population of NMDARs, such as synaptic ones, enabling the selective activation of a different population, such as extrasynaptic NMDARs. However, the usefulness of this approach is dependent on the stability of MK-801 blockade after washout. We have revisited this issue, and confirm that recovery of NMDAR currents from MK-801 blockade is enhanced by channel opening by NMDA, and find that it is further increased when Mg^2+^ is also present. In the presence of Mg^2+^, 50% recovery from MK-801 blockade is achieved after 10′ of 100 μM NMDA, or 30′ of 15 μM NMDA exposure. In Mg^2+^-free medium, NMDA-induced MK-801 dissociation was found to be much slower. Memantine, another PCP-site antagonist, could substitute for Mg^2+^ in accelerating the unblock of MK-801 in the presence of NMDA. This suggests a model whereby, upon dissociation from its binding site in the pore, MK-801 is able to re-bind in a process antagonized by Mg^2+^ or another PCP-site antagonist. Finally we show that even when all NMDARs are pre-blocked by MK-801, incubation of neurons with 100 μM NMDA in the presence of Mg^2+^ for 2.5 h triggers sufficient unblocking to kill >80% of neurons. We conclude that while synaptic MK-801 ‘pre-block’ protocols are useful for pharmacologically assessing synaptic vs. extrasynaptic contributions to NMDAR currents, or studying short-term effects, it is problematic to use this technique to attempt to study the effects of long-term selective extrasynaptic NMDAR activation.

This article is part of the Special Issue entitled ‘Glutamate Receptor-Dependent Synaptic Plasticity’.

## Highlights

► Use-dependent recovery of NMDAR currents after MK-801 blockade is boosted by Mg^2+^. ► Use-dependent recovery is also accelerated by memantine. ► NMDA + Mg^2+^ can trigger 45% MK-801 dissociation in only 10 min ► NMDA + Mg^2+^ can promote death in neurons with NMDARs 100% pre-blocked by MK-801. ► Care should be taken not to overestimate the stability of MK-801 blockade of NMDARs.

## 1 Introduction

MK-801 (dizocilpine), a compound originally characterised as an anti-convulsant drug, is a potent NMDAR antagonist (Wong et al., 1986). MK-801 binds selectively and with high affinity to NMDARs when they are in their open state (Huettner and Bean, 1988) and as such is referred to as a use-dependent or open-channel blocker.

Early electrophysiological and ligand binding studies revealed that the blockade of NMDARs by MK-801 persisted long after the drug had been washed out (Huettner and Bean, 1988; Reynolds and Miller, 1988). This unusual property meant that MK-801 blockade of NMDARs is highly stable and could be regarded as “irreversible” over the timecourse of many experiments, leading to it being used to “permanently” block a sub-population of NMDARs in order to study a separate population of non-blocked NMDARs. An example of such use is in the study of synaptic and extrasynaptic NMDARs (Hardingham et al., 2002; Tovar and Westbrook, 1999). Differential signalling by the neuroprotective phasic activation of synaptic NMDARs compared to the deleterious tonic activation of extrasynaptic NMDARs, is a topic of ongoing interest (Bordji et al., 2010; Dick and Bading, 2010; Hardingham and Bading, 2002, 2010; Ivanov et al., 2006; Leveille et al., 2008; Milnerwood et al., 2010; Okamoto et al., 2009; Papadia et al., 2008; Soriano et al., 2009; Tu et al., 2010; Wahl et al., 2009; Zhang et al., 2007).

In order to selectively activate extrasynaptic NMDARs, synaptic NMDARs must first be pre-blocked by, for example, driving synaptic activity in the presence of MK-801, leaving extrasynaptic NMDARs unaffected. Following washout, MK-801 mediated block of synaptic NMDARs persists, enabling the selective activation of extrasynaptic NMDARs simply by bath-applying an NMDAR agonist (Bengtson et al., 2008; Hardingham et al., 2002). The same technique has also been employed to other ends, such as the analysis of recovery of synaptic currents as an indicator of NMDAR diffusion from extrasynaptic to synaptic sites (Tovar and Westbrook, 2002).

While the stability of the MK-801 blockade means that it can be relied upon to largely persist for the timecourse of short-term experiments, it is important to define precisely how persistent MK-801 blockade is. For example, a recent study involved an attempt to selectively activate extrasynaptic NMDARs for 150 min by relying on the MK-801 mediated pre-block of synaptic NMDARs remaining over this time period (Wroge et al., 2012). However, it is clear, even from early experiments, that MK-801 blockade is not irreversible and moreover, that its stability depends on the activation state of the receptor. In particular, dissociation of MK-801 from the NMDAR is increased when the receptor is in the open state: it was observed originally that recovery from MK-801 blockade could be accelerated by NMDA, i.e. that recovery is use-dependent (Huettner and Bean, 1988). However, in that particular study the authors reported a time constant for recovery of 92 ± 106 min (mean ± SD) reflecting the fact that recovery was observed in several cells but not others, the reason for which is unclear. Others also reported observing use-dependent recovery from MK-801 blockade using techniques such as radio-ligand binding and Ca^2+^ imaging (Reynolds and Miller, 1988; Yuzaki et al., 1990). However, Ca^2+^ imaging experiments are indirect indicators of NMDAR current and ligand binding assays often involve the use of membrane preparations that are by definition not at physiological membrane potential. Another issue that requires clarification is the influence of Mg^2+^ on the stability of the MK-801 block. Ca^2+^ imaging studies suggest that on its own Mg^2+^ does not promote recovery of NMDARs from MK-801 blockade (Yuzaki et al., 1990). However, we wanted to determine the influence of Mg^2+^ on the use-dependent recovery of NMDAR currents from MK-801. Mg^2+^ also binds the pore of the NMDAR, and under physiological conditions its voltage-dependent unbinding is essential for full channel activity. Moreover, Mg^2+^ has been reported to inhibit the binding of radiolabelled forms of MK-801 (Hubbard et al., 1989; Reynolds and Miller, 1988) suggestive of mutually antagonistic binding. Here we have revisited the issue of the stability of MK-801 blockade, focussing in particular on the extent to which NMDAR currents recover from MK-801 blockade under conditions of agonist exposure, both in the presence and absence of extracellular Mg^2+^.

## 2 Material and methods

### 2.1 Neuronal culture and assessment of cell death

Cortical rat neurons were cultured as described (Al-Mubarak et al., 2009; Soriano et al., 2009) at a density of between 9 and 13 × 10^4^ neurons per cm^2^ from E21 rats with Neurobasal A growth medium supplemented with B27 (Invitrogen, Paisley, UK). Stimulations of cultured neurons were done in most cases after a culturing period of 8–11 days during which neurons develop a network of processes, express functional NMDA-type and AMPA/kainate-type glutamate receptors, and form synaptic contacts (Papadia et al., 2008). To elicit an excitotoxic insult, neurons were first placed overnight into a minimal defined medium (Papadia et al., 2005) containing 10% MEM (Invitrogen), 90% Salt-Glucose-Glycine (SGG) medium ((Bading et al., 1993); SGG: 114 mM NaCl, 0.219% NaHCO_3_, 5.292 mM KCl, 1 mM MgCl_2_, 2 mM CaCl_2_, 10 mM HEPES, 1 mM Glycine, 30 mM Glucose, 0.5 mM sodium pyruvate, 0.1% Phenol Red; osmolarity 325 mosm/l, (Papadia et al., 2005)). NMDA and MK-801 were from Tocris Bioscience, Bristol, UK. To assess cell death, neurons were fixed and subjected to DAPI staining and cell death quantified by counting (blind) the number of shrunken, pyknotic nuclei as a percentage of the total.

### 2.2 Electrophysiological recording and analysis and assessment of MK-801 unblocking

**Fig. 1:**
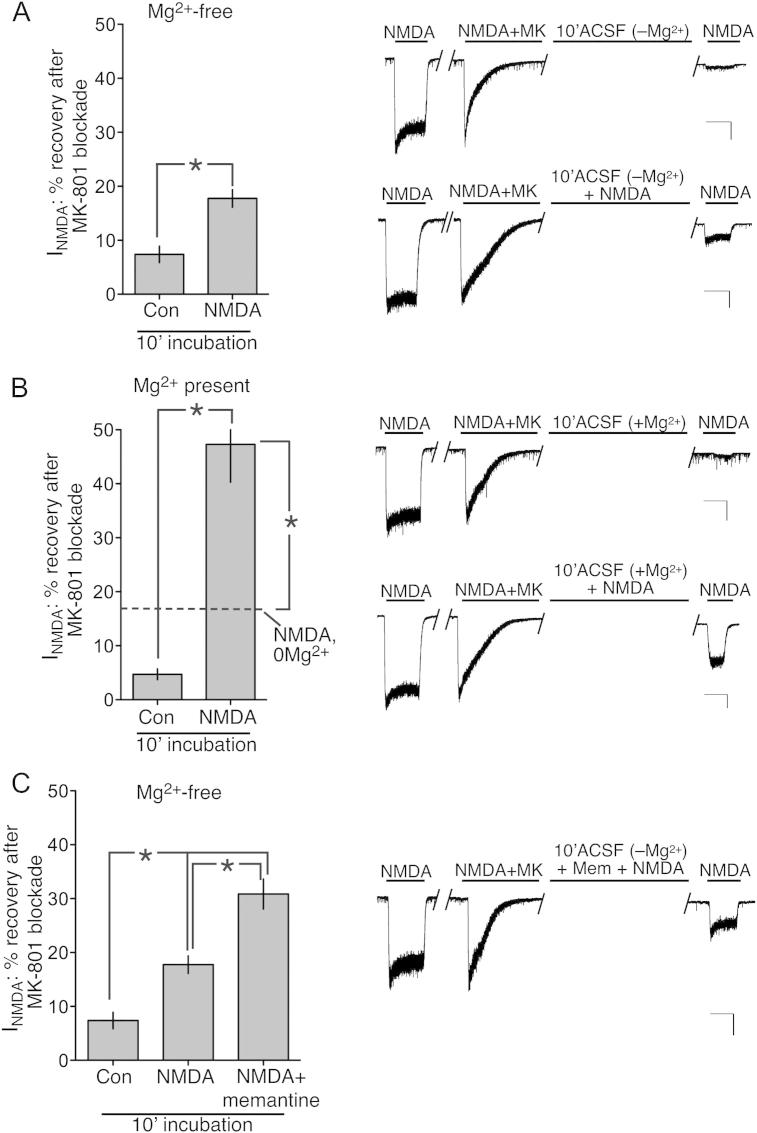
Both Mg^2+^ and memantine accelerate recovery from MK-801 blockade of NMDARs in the presence of agonist (NMDA). A) Confirmation of agonist-dependent dissociation of MK-801 from NMDARs. Whole-cell NMDAR currents evoked by 100 μM NMDA were recorded, after which all NMDARs were blocked by co-application of NMDA + MK-801 (MK). Neurons were then held at −60 mV and continuously perfused for 10 min in ACSF ± NMDA (zero Mg^2+^), after which whole-cell currents were once again recorded and expressed as a % of the original current. *p < 0.05, Student unpaired *t*-test (*n* = 8 Con, *n* = 9 NMDA). Examples traces are shown to the right. Scale bar = 5 s (*x*-axis), 200 pA (*y*-axis) here and throughout Fig. 1. B) Agonist-dependent recovery of NMDAR currents from MK-801 blockade is accelerated by Mg^2+^. Neurons were subjected to exactly the same procedure as (A) except that the 10 min incubation period ± NMDA was carried out in Mg^2+^-containing ACSF (1 mM MgCl_2_). Dotted line shows the NMDA-induced unblocking level in the *absence* of Mg^2+^. *p < 0.05, Student unpaired *t*-test (*n* = 7 Con, *n* = 8 NMDA). C) Agonist-dependent recovery of NMDAR currents from MK-801 blockade is accelerated by the antagonist memantine. Where indicated, memantine (Mem, 10 μM) was included in the 10 min recovery solution. *p < 0.05, Student unpaired *t*-test (*n* = 7 Con, *n* = 8 NMDA, *n* = 10 NMDA + memantine).

**Fig. 2:**
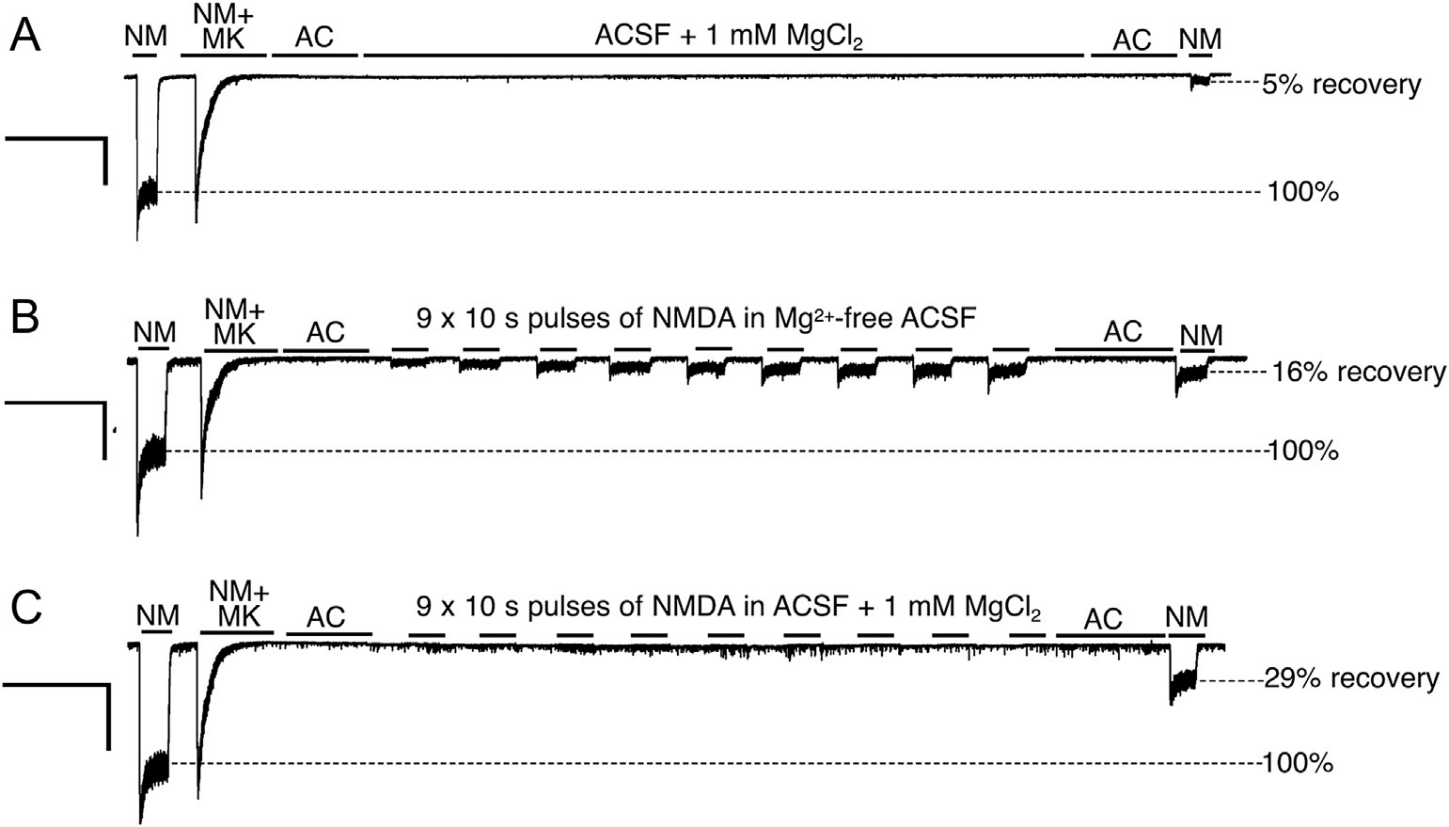
Illustration of the influence of repeated applications of NMDA ± Mg^2+^ on recovery from MK-801 blockade. Example traces further illustrating the agonist and Mg^2+^-dependent recovery of NMDAR currents from MK-801 blockade. Following MK-801 pre-block of NMDAR currents, neurons were subjected to either a period of washout in ACSF + Mg^2+^ (A) or to 9 × 10 s applications of NMDA ± Mg^2+^ (B, C), after which whole-cell currents were measured and the % recovery calculated. Scale bars refer to 25 s (*x* axis) and 400 pA (*y* axis). NM = NMDA; MK = MK-801; AC = ACSF.

**Fig. 3:**
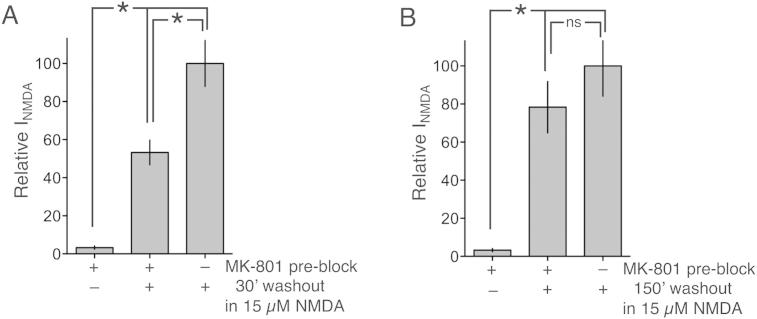
Studying the recovery of NMDAR currents from MK-801 in unclamped neurons. A) Where indicated, all NMDARs were pre-blocked by the addition of 100 μM NMDA + 10 μM MK-801. After extensive washing in which coverslips from all groups including those without pre-block were transferred 5 times into fresh media, the neurons were incubated for 30 min in Mg^2+^-containing medium in the presence of a sub-toxic dose of NMDA (15 μM). After this period, NMDAR whole-cell currents were measured as previously. Currents were compared to levels recorded in neurons treated identically except that they were not subjected to MK-801 pre-block. *p < 0.05, Student unpaired *t*-test (*n* = 7 in all conditions). B) Neurons were treated as in (A) except that the incubation/washout period was for 150 min *p < 0.05, Student unpaired *t*-test (*n* = 7 in all conditions).

**Fig. 4:**
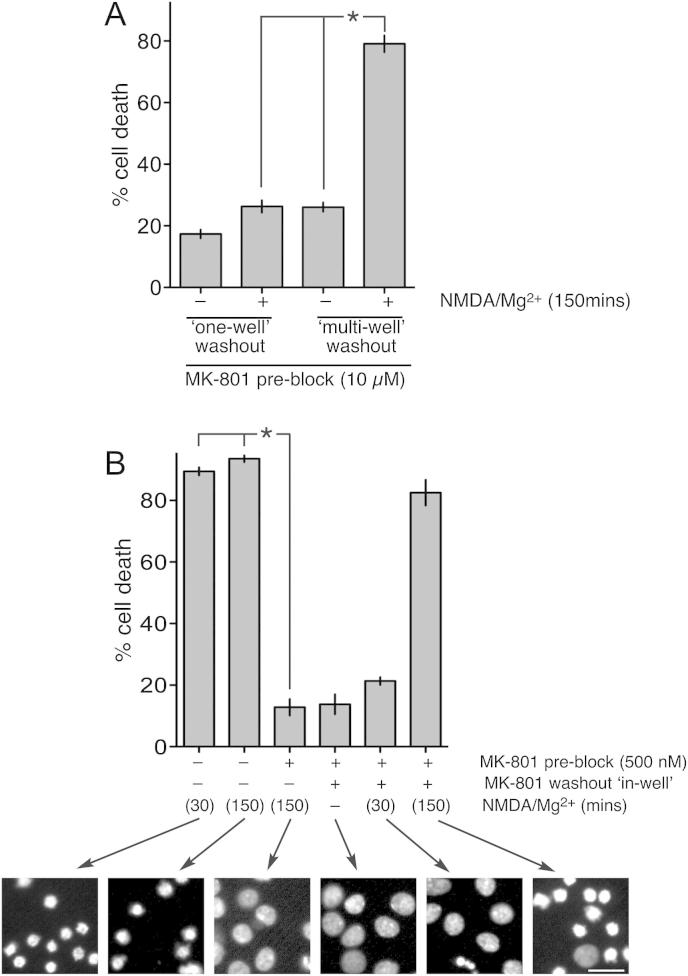
Agonist-induced recovery from MK-801 blockade can lead to excitotoxic cell death even when 100% of NMDARs are pre-blocked. A) NMDARs were pre-blocked by the application of 100 μM NMDA + 10 μM MK-801 for 10 min. MK-801 was then washed out either by 5 sequential fresh medium changes within the same well (‘one-well’ wash) or by the transfer of coverslips containing neurons sequentially through 5 wells containing fresh medium (‘multi-well’ wash). After this wash period, neurons were exposed, where indicated, to 100 μM NMDA for 150 min, after which all NMDARs were re-blocked by the addition of MK-801. 24 h after the commencement of the experiment, cells were fixed and cell death analysed. B) Where indicated, NMDARs were pre-blocked by the application of 100 μM NMDA + 500 nM MK-801 for 10 min. Where performed, washout involved 5 sequential fresh medium changes within the same well (‘one-well’ wash). After this wash period, neurons were exposed, where indicated, to 100 μM NMDA for either 30 or 150 min, after which all NMDARs were re-blocked by the addition of MK-801. 24 h after the commencement of the experiment, cells were fixed and cell death analysed. Lower figure shows example pictures (scale bar = 25 μm).

Whole-cell NMDA-evoked currents in cultured rat cortical neurons were recorded using an Axopatch 200B amplifier (Molecular Devices) using patch-pipettes made from thick-walled borosilicate glass with a tip resistance of 4–8 MΩ, which were filled with an ‘internal’ solution that contained (in mM) potassium gluconate 141, NaCl 2.5, HEPES 10, EGTA 11; pH 7.3 with KOH. Experiments were conducted at room temperature (18–21 °C) in an Mg^2+^-free external/ACSF solution containing (in mM): NaCl 150, KCl 2.8, HEPES 10, CaCl_2_ 2, glucose 10, EDTA 0.01; pH to 7.3 with NaOH. Tetrodotoxin (300 nM) was included to block action potential-driven excitatory postsynaptic events. Whole-cell NMDA currents were elicited by bath perfusion of NMDA (100 μM, unless otherwise stated) in the presence of glycine (100 μM). Access resistances were monitored and, recordings where this changed by >20% were discarded. Neurons were voltage-clamped at −60 mV. Currents were filtered at 2 kHz and digitized at 5 kHz via a BNC-2090A/PCI-6251 DAQ board interface (National Instruments, Austin, TX) and analysed using WinEDR software (Dr John Dempster, University of Strathclyde, Glasgow,UK). To pre-block all NMDARs, neurons were exposed to 100 μM NMDA in the presence of MK-801 (use at 10 μM except in Fig. 4b where 500 nM was used). The washout protocols for Figs. 1 and 2 involved constant perfusion of the neurons with media of different compositions (see legends). The washout protocol for Fig. 3 involved the transfer of coverslips containing neurons sequentially through 5 wells containing fresh medium (‘multi-well’ wash). The washout protocols for Fig. 4 involved the multi-well wash but also a ‘one-well’ wash protocol whereby the neurons were washed with 5 sequential fresh medium changes within the same well. In Figs. 1 and 2, absolute measurements of MK-801 unblocking were determined by comparing whole-cell NMDAR currents pre- and post- MK-801 blockade in a single neuron. In Fig. 3, the level of unblocking was estimated by comparing whole-cell currents post-washout to NMDAR currents in parallel sister coverslips that had not been subjected to MK-801 pre-blockade.

### 2.3 Induction of excitotoxicity

An approach similar to those previously reported was employed (Soriano et al., 2006). To induce neuronal death, neurons were exposed to NMDA (100 μM). To terminate NMDAR activation MK-801 (10 μM) was added at the desired time point. Exposure to excitotoxic concentrations of NMDA or glutamate leads to neurons displaying swollen cell bodies and pyknotic nuclei with small irregular chromatin inclusions. Such characteristics are indicative of necrotic, as opposed to apoptotic, cell death (Martel et al., 2012). Assessment of cell death was made 24 h after exposure to NMDA by calculating the ratio of 4′, 6-diamidino-2-phenyl indole-stained pyknotic nuclei as a percentage of the total nuclei.

## 3 Results and discussion

Whole-cell NMDAR currents were recorded at −60 mV prior to blockade of all NMDARs by the application of NMDA in the presence of MK-801. After MK-801 wash-out, the % recovery of NMDAR currents from MK-801 blockade was studied after 10 min exposure to various conditions. Consistent with previous studies, MK-801 blockade was stable in normal Mg^2+^-free ACSF: only 7% recovery was observed (Fig. 1a). Original studies showed that recovery from MK-801 blockade could be accelerated by NMDA (Huettner and Bean, 1988), i.e. that recovery is use-dependent. However, as stated in the Introduction, the large standard deviation for recovery time reflected the fact that recovery was observed in several cells but not others (potentially due to incomplete washout of MK-801). Others have also reported use-dependent recovery using techniques such as radio-ligand binding and Ca^2+^ imaging (Reynolds and Miller, 1988; Yuzaki et al., 1990). We observed that 10 min exposure to 100 μM NMDA promoted an approximate 20% recovery of NMDAR currents from MK-801 blockade (Fig. 1a), confirming the use-dependent nature of the unblock and consistent with the model that the NMDAR must be open for MK-801 to leave the channel pore.

We next turned our attention to the influence of Mg^2+^ on the dissociation of MK-801 from NMDARs. Ligand binding studies have suggested that Mg^2+^ may accelerate its dissociation (Reynolds and Miller, 1988), however other studies (employing Ca^2+^ imaging approaches) have found no affect of Mg^2+^; (Yuzaki et al., 1990). We observed that 10 min exposure in 1 mM Mg^2+^-containing ACSF failed to promote appreciable recovery of NMDAR currents from MK-801 blockade (Fig. 1b), consistent with (Yuzaki et al., 1990) but contrary to the ligand binding study (Reynolds and Miller, 1988). We next investigated the effect of Mg^2+^ on NMDA-induced recovery from MK-801 blockade. Interestingly, Mg^2+^ strongly increased the level of MK-801 unblock in the presence of NMDA: 10 min exposure to 100 μM NMDA + 1 mM Mg^2+^ led to recovery of around 50% of the original whole-cell NMDAR current (Fig. 1b). Thus, a combination of the channel in an open activated state, together with Mg^2+^, promotes recovery of NMDAR currents from MK-801 blockade. This is consistent with the long-held notion that MK-801 dissociation is only possible from an open NMDAR. However the effect of Mg^2+^ suggests a model whereby either Mg^2+^ promotes MK-801 dissociation or that, upon dissociation from its binding site in the pore, MK-801 is able to re-bind in a process antagonized by Mg^2+^. The effect of Mg^2+^ also raises the possibility that other NMDAR antagonists that act at this site could also promote the loss of MK-801 from an open receptor. We investigated this by replacing Mg^2+^ in our MK-801 dissociation experiments with memantine, another “PCP site” antagonist. As in previous experiments, all NMDARs were pre-blocked by the application of NMDA in the presence of MK-801. After MK-801 wash-out, the % recovery of NMDAR currents from MK-801 blockade was studied after 9 min exposure to NMDA + memantine, followed by an additional minute to permit washout of memantine (which dissociates quickly from the NMDAR unlike MK-801 (Chen and Lipton, 1997; Parsons et al., 1995)). We found that, as was the case with Mg^2+^ and in the presence of NMDA, memantine accelerated the recovery of NMDAR currents from MK-801 blockade (Fig. 1c). We conclude that when MK-801 has dissociated from its site in the pore, that there is a finite chance that it rebinds its site rather than leave the pore entirely and further, that the effect of enhanced MK-801 unblock in the presence of either Mg^2+^ or memantine is due to these channel blockers preventing the re-binding of MK-801.

The effect of Mg^2+^ on NMDA-induced unblocking of the NMDAR can be visualized in real time by studying the effect of repeated applications of NMDA ± Mg^2+^ on NMDAR current in neurons pre-blocked by MK-801 (Fig. 2). Nine periods of 10 s exposures to 100 μM NMDA in zero Mg^2+^ promotes some use-dependent recovery from MK-801 blockade, compared to ACSF alone (Fig. 2a,b). Because Mg^2+^ is absent, the incremental increases in NMDAR currents as MK-801 dissociates can be visualized. In contrast, when this experiment was repeated in the presence of Mg^2+^, the repeated NMDA applications did not elicit measurable currents because under voltage clamp at −60 mV, Mg^2+^ blocks NMDARs even when they are free of MK-801. However, the extent of the recovery from MK-801 blockade becomes apparent when whole-cell currents are measured in the absence of Mg^2+^: a 30% recovery from the MK-801 block was observed (Fig. 2c).

We next studied the influence of NMDA plus Mg^2+^ on recovery of NMDAR currents from MK-801 blockade in unclamped neurons in culture. All NMDARs were pre-blocked by the addition of 100 μM NMDA + 10 μM MK-801 for 3 min, followed by five medium changes to remove MK-801, which involved transferring the coverslips sequentially along a row of 5 wells containing fresh medium. NMDA + Mg^2+^ was then added to the neurons for either 30 min or 150 min, after which cells were patched and whole-cell currents recorded. We used a low, non-toxic concentration of NMDA (15 μM) because, even though this would likely promote a lower level of use-dependent unblocking, we wanted to ensure that the neurons would not die if MK-801 dissociated from a substantial proportion of NMDARs. We found that, after 30 min of exposure to 15 μM NMDA in the presence of Mg^2+^, NMDAR currents had recovered to around half of that in control (non-pre-blocked) cells (Fig. 3a). After 150 min, currents were not significantly different from control (Fig. 3b). Thus, in unclamped neurons in Mg^2+^-containing medium, appreciable recovery of NMDAR currents following MK-801 pre-block is observed over tens of minutes, even when sub-maximal concentrations of agonist are employed.

This result indicates that if the above experiment was performed with toxic NMDA concentration, that sufficient unblocking of the NMDAR would take place to kill neurons. To study this, all NMDARs were again pre-blocked by the addition of 100 μM NMDA + 10 μM MK-801 for 10 min (Fig. 4a). Washout involved either five fresh medium changes within the same well (‘one-well’ washing), or the transfer of coverslips containing the neurons sequentially through a row of wells each containing fresh medium (‘multi-well’ washing). Different washout approaches were used because, while washing within the same well was easiest, we felt that washing in multiple wells was potentially more thorough and mirrored the electrophysiology experiments exactly. Following washout, NMDA was applied at 100 μM in the presence of Mg^2+^ for 150 min, after which more MK-801 was added (to terminate NMDA-induced activity). Cell death was assessed 24 h later to determine whether the period of NMDA/Mg^2+^ incubation was sufficient to cause enough dissociation of MK-801 from NMDARs to promote NMDAR-dependent cell death. Unlike in previous experiments, we did not compare NMDA/Mg^2+^ incubation to NMDA/zero Mg^2+^. This is because, while there would be less MK-801 unblocking in the absence of Mg^2+^, this smaller number of unblocked channels would pass more current than if Mg^2+^ were present, making it difficult to interpret any differences in levels of cell death in the presence vs. absence of Mg^2+^. We observed that in neurons where NMDARs were pre-blocked and MK-801 washout involved their transfer through five wells of fresh medium, a 150 min exposure of 100 μM NMDA/Mg^2+^ promoted NMDAR-dependent death in around 80% of neurons (Fig. 4a).

Thus, following pre-block of all NMDARs and after the thorough ‘multi-well’ wash procedure, MK-801 unblocks sufficiently in the presence of NMDA and Mg^2+^ over 150 min to kill neurons. Importantly, significant NMDA-induced cell death was *not* observed in the ‘one-well’ washing protocol, suggesting that this protocol may be less effective at removing all MK-801. Since MK-801 has a reported K_d_ of 3–30 nM (Berman and Murray, 1996; Reynolds and Miller, 1988; Wong et al., 1986), functionally relevant concentrations of MK-801 could remain even when washing leaves as little as 0.1% of the starting concentration (10 μM) behind. We reasoned that the ‘one-well’ washing protocol may be sufficient if the initial concentration of MK-801 used was reduced from 10 μM to a still-effective, but lower concentration. Thus, we repeated the experiment, using MK-801 to pre-block at 500 nM, rather than 10 μM (Fig. 4b). Under these conditions, MK-801 still effectively blocked NMDAR currents (data not shown) and prevented excitotoxicity when left in the well (Fig. 3b) but this time the ‘one-well’ washout protocol was effective in removing excess MK-801, and excitotoxicity due to MK-801 unblock was clearly observed (Fig. 4b). Collectively these experiments highlight how difficult thorough MK-801 wash-out is to achieve, potentially leading to erroneous neuroprotective effects if washout is imperfect. That said, if washout is thorough, it is clear that when all NMDARs are pre-blocked, MK-801 unblocking from NMDARs under conditions of agonist exposure occurs sufficiently over a 150 min timescale to cause cell death.

As stated in the Introduction, the very slow off-rate of MK-801 has been exploited to use-dependently selectively pre-block synaptic NMDARs, enabling the selective activation of extrasynaptic NMDARs following MK-801 washout. Tovar and Westbrook were among the first to use MK-801 to isolate extrasynaptic NMDARs (Tovar and Westbrook, 1999). Using autaptic micro-island cultures, they could stimulate action potential firing by brief depolarization of the neuron, resulting in an autaptic EPSC due to the trans-synaptic activation of synaptic AMPA and NMDARs. By stimulating autaptic EPSCs in the presence of MK-801, the authors were able to exploit the use-dependency of MK-801 to selectively block those NMDARs activated by the stimulation protocol. In other words, synaptic, but not extrasynaptic NMDARs became progressively blocked over the course of multiple stimulations. In order to selectively activate extrasynaptic NMDARs they exploited the very slow off-rate of MK-801: following open channel blockade of synaptic NMDARs, MK-801 was removed from the recording medium, leaving synaptic NMDARs blocked. Under these conditions, bath application of NMDA simply results in the activation of extrasynaptic NMDARs (Tovar and Westbrook, 1999). By combining this approach with other pharmacological tools the authors reported some differences in NMDAR subunit composition at synaptic vs. extrasynaptic locations (Tovar and Westbrook, 1999).

Other variations on this approach have been employed to selectively activate extrasynaptic NMDARs, using different initial methods to promote selective synaptic NMDAR stimulation. Examples include inducing action potential bursting in neuronal cultures with the GABA_A_ receptor antagonist bicuculline (Hardingham et al., 2002). We used this technique to show that selective chronic activation of extrasynaptic NMDARs results in rapid (seconds time-scale) perturbation of mitochondrial membrane potential, an early event in excitotoxicity which precedes and predicts cell death (Hardingham et al., 2002). Similar protocols have also used MK-801 with concurrent synaptic stimulation to isolate the extrasynaptic NMDA receptor pool in brain slices (Harris and Pettit, 2007).

An alternative to the bicuculline method is simply to allow spontaneous quantal release of glutamate to activate synaptic NMDARs by including TTX and zero Mg^2+^ in the medium (Martel et al., 2009; Nakayama et al., 2005; Papadia et al., 2008; Puddifoot et al., 2012). If MK-801 is also present this results in the progressive blockade of synaptic NMDARs over about 10 min and has been employed by us to study the control of the relative proportion of synaptic vs. extrasynaptic NMDARs and their subunit composition (Martel et al., 2009, 2012; Papadia et al., 2008). It should be emphasized that all protocols to isolate synaptic or extrasynaptic NMDARs must be validated in the system under study. For example, in acute slices the tonic activation of extrasynaptic NMDARs by ambient glutamate of non-synaptic origin means that MK-801 application in a cell clamped at +40 mV can result in the use-dependent blocking of *extra*synaptic NMDARs (Le Meur et al., 2007).

Another issue is the strength of the protocol used for triggering synaptic activity. While stronger protocols for driving synaptic activity have been employed, they risk activating extrasynaptic NMDARs as well, either via glutamate spillover or by imposing conditions that promote activation of extrasynaptic NMDARs by low level ambient glutamate (such as Mg^2+^-free medium). Indeed, a recently described protocol involving bicuculline treatment in *Mg*^*2+*^*-free* medium (Wroge et al., 2012) in our hands blocked 98.1 ± 1.1% of all NMDARs (*n* = 7).

An implication of the current study is that while the above pre-block protocols remain valid for the short term experiments described above, it may be problematic to rely on continued MK-801 blockade of synaptic NMDARs under continuous agonist exposure as a strategy to study *prolonged* selective extrasynaptic NMDAR activation. Experiments involving up to 150 min of continuous agonist exposure (in the presence of Mg^2+^) have been employed (Wroge et al., 2012), at which time, according to our study, MK-801 would be substantially absent from synaptic NMDARs. Thus, care should be taken not to take the “irreversible” nature of MK-801 blockade too literally.
